# Fellow-Workers and Their Doings

**Published:** 1914-08-08

**Authors:** 


### ROUND THE WORLD.

### Fellow-Workers and their Doings.

[The Editor Invites Early Information and Contributions Marked "Fellow-Workers."]

Dr. Charles Miller, who in an interesting communication to the *Lancet* (July 25, 1914) draws attention to the possible dangers of indiscriminately injecting tuberculin in cases of consumption where the disease has apparently been arrested by other treatment, is one of the junior members of the medical staff at the London Hospital. It is not improbable that the sad case he cites of a woman patient who was compelled by an official fiat in connection with the Insurance Act to undergo tuberculin treatment, although apparently recovered from pulmonary tuberculosis, does not stand alone; consequently Dr. Miller's warning against the activities of some tuberculin "enthusiasts" is by no means inept.

Well-known authorities on special subjects do not always choose for official addresses matters of which they are most cognisant, but it may be hoped that Sir Ronald Ross will deal with some aspects of tropical medicine that will be of assistance to the student when he fulfils his present promise to give the opening Huxley Lecture at Charing Cross Hospital Medical School in October. Few workers have done so much to advance the cause of health in hot countries and so to pave the way for the free habitation of white men in our tropical dependencies as Sir Ronald Ross, whose public services received recognition by the conferment of a K.C.B. three years ago. Formerly a student at St. Bartholomew's, he found his way to the field of research that has made him famous through the Indian Medical Service, from which he retired some years ago with the rank of major. A number of academic distinctions have been showered upon him since his qualification as a member of the Royal College of Surgeons in 1879.

Captain W. P. MacArthur, R.A.M.C., has been working at the vaccine treatment of enteric fever, in connection with which he is able to report very favourably. In a communication to the *British Medical Journal* (July 25, 1914) dealing with what is said to be the first series of cases of typhoid vaccine therapy published from Mauritius, this investigator records striking successes, particularly where the treatment was begun in the early stages of the illness. Of sixty-three cases fifty-two were treated early and eleven late, and, with the exception of one fatality of broncho-pneumonia, all complications and relapses occurred in the late series. Captain MacArthur, who is an M.D., F.R.C.P.I., points out that in his experience the injection rendered the disease definitely milder, whilst most of the distressing symptoms quickly disappeared. The dosage given varied from 150 to 300 million, increased every two or three days.

Dr. Geoffrey Viner, who has been appointed assistant surgeon to the Royal Westminster Ophthalmic Hospital, has lately been fulfilling the duties of chief assistant there. Dr. Viner is a St. Bartholomew's man, and held several resident appointments at his old hospital, including that of ophthalmic house surgeon. Having become an M.D. London and F.R.C.S. Eng., he settled in ophthalmic practice in the West End, having been appointed clinical assistant to the Royal London Ophthalmic Hospital and ophthalmic clinical assistant at "Bart's." Last year he contributed an article on the significance of ocular palsies to the *St. Bartholomew's Hospital Journal*.

Sir John Bland-Sutton, whose interesting address to the British Medical Association on "The Surgeon of the Future" attracted much attention, has had a remarkable career as an operating surgeon and hospital benefactor. Not only has he stamped his personality on the surgical work at the "Middlesex," but he will be remembered at that institution as a generous supporter of new developments, particularly in connection with pathological research. This distinguished operator is a man of wide tastes, and his replica of a Persian hall in his Brook Street house has earned the admiration of many. Entering the Middlesex Hospital in 1878, Sir John steadily climbed the staff ladder and rapidly made a name for himself, both as a practical surgeon and as an authoritative teacher.

Dr. A. E. Garrod, who delivered the corresponding address in medicine, is a well-known authority on disorders of metabolism, particularly in their relation to joint troubles. His subject, "Medicines from the Chemical Standpoint," suggested what will probably be the treatment of the future. There seems little doubt that the next great advance in physical therapeutics will be attained through a close study of bio-chemistry aided by the elaboration of synthetic remedies such as have already been manufactured with success by Ehrlich and his followers. Dr. Garrod, who is an M.D., F.R.C.P., and F.R.S., is physician and lecturer on chemical pathology at St. Bartholomew's, as well as physician to the Hospital for Sick Children in Great Ormond Street.

Dr. James Niven, who has been lately awarded a gold medal by the Royal Institute of Public Health, has greatly aided the cause of preventive medicine by his work. He is, of course, medical officer of health for Manchester. Qualifying M.B. Cantab. from St. Thomas's in 1880, Dr. Niven soon devoted himself to public-health problems, in connection with which he obtained important appointments. Much of his recent work has been carried out in association with the anti-consumption campaign, some of his most practical writings having dealt with matters cognate thereto.

Professor J. C. Bose's lecture, promised for October, on "The Modification of Response in Plants Under the Action of Drugs," may be looked forward to as an interesting event. This well-known investigator has attacked numerous problems of biology, particularly in regard to electrical action and response, that have an important bearing on medical work. Educated at Calcutta and Christ's College, Cambridge, Professor Jagadis Chandra Bose became an M.A. Cantab. and D.Sc. London. Now occupying an important chair in the Presidency College, Calcutta, his work has received public recognition by the confirmation of a C.I.E. and C.S.I.

Dr. T. B. Johnston, who has been appointed to the new post of lecturer and demonstrator in anatomy at University College, London, is an Edinburgh student who took the M.B., Ch.B. of his University in 1906; since when he has pursued the special subject of anatomy and held the important post of lecturer thereon at Edinburgh University. Before settling down to anatomical study Dr. Johnston held the appointments of house surgeon to the Ramsgate General Hospital and visiting surgeon to the Ramsgate and St. Lawrence Dispensary. He has investigated and written on various rare anatomical findings in the human body.

